# Production of Algal Biomass and High-Value Compounds Mediated by Interaction of Microalgal *Oocystis* sp. KNUA044 and Bacterium *Sphingomonas* KNU100

**DOI:** 10.4014/jmb.2009.09055

**Published:** 2020-12-14

**Authors:** Ho Na, Seung-Woo Jo, Jeong-Mi Do, Il-Sup Kim, Ho-Sung Yoon

**Affiliations:** 1 Department of Biology, Kyungpook National University, Daegu 41566, Republic of Korea; 2 School of Life Sciences, BK21 Plus KNU Creative BioResearch Group, Kyungpook National University, Daegu 41566, Republic of Korea; 3 Advanced Bio-Resource Research Center, Kyungpook National University, Daegu 41566, Republic of Korea

**Keywords:** *Oocystis* spp., *Sphingomonas* spp., symbiotic interaction, algal biomass, eicosapentaenoic acid, fucose

## Abstract

There is growing interest in the production of microalgae-based, high-value by-products as an emerging green biotechnology. However, a cultivation platform for *Oocystis* sp. has yet to be established. We therefore examined the effects of bacterial culture additions on the growth and production of valuable compounds of the microalgal strain *Oocystis* sp. KNUA044, isolated from a locally adapted region in Korea. The strain grew only in the presence of a clear supernatant of *Sphingomonas* sp. KNU100 culture solution and generated 28.57 mg/l/d of biomass productivity. Protein content (43.9 wt%) was approximately two-fold higher than carbohydrate content (29.4 wt%) and lipid content (13.9 wt%). *Oocystis* sp. KNUA044 produced the monosaccharide fucose (33 μg/mg and 0.94 mg/l/d), reported here for the first time. Fatty acid profiling showed high accumulation (over 60%) of polyunsaturated fatty acids (PUFAs) compared to saturated (29.4%) and monounsaturated fatty acids (9.9%) under the same culture conditions. Of these PUFAs, the algal strain produced the highest concentration of linolenic acid (C18:3 ω3; 40.2%) in the omega–3 family and generated eicosapentaenoic acid (C20:5 ω3; 6.0%), also known as EPA. Based on these results, we suggest that the application of *Sphingomonas* sp. KNU100 for strain-dependent cultivation of *Oocystis* sp. KNUA044 holds future promise as a bioprocess capable of increasing algal biomass and high-value bioactive by-products, including fucose and PUFAs such as linolenic acid and EPA.

## Introduction

Microalgae, photosynthetic organisms that capture carbon dioxide, can produce various antioxidants and pigments, such as carotenoids, vitamins, carbohydrates, lipids (including polyunsaturated fatty acids), and proteins (including the essential amino acids methionine, threonine, and tryptophan) [1-3]. They have a wide range of applications in the biofuel, food, feed, agriculture, cosmetics, and pharmaceutical industries [1-3]. Microalgae are useful in aquaculture as a source of biomolecules and biomass that can improve the nutritional value of food or provide additional health benefits [4]. Particularly, for food, aquaculture, and healthcare applications, it is important to select and use microalgal species and strains that will enhance the production of the desired compounds [5]. It is therefore necessary to explore a wider and more diverse pool of microalgae strains [5]. Moreover, for economical production, it is desirable to derive multiple products such as lipids and high value-added by-products from the same biomass in one growth cycle [6]. Furthermore, optimizing culture conditions by selecting organisms that can overcome limitations imposed by ambient conditions and selecting strains with high lipid and protein content can also lower the unit cost for microalgae-based industries such as biofuels and food [2,6,7].

*Oocystaceae* is a monophyletic family in the class Trebouxiophyceae, phylum Chlorophyta, which can be identified by the ultrastructure of its cell wall at the molecular level [8-10]. However, the morphology of genera and species in this family remains unclear [8-10]. *Oocystaceae*, especially genus *Oocystis*, are generally considered common freshwater coccal microalgae with a distinctive morphology of oval or fusiform cells, usually persisting within several layers of a mother cell wall for a long time [10, 11]. This family is widely considered distinct, with a characteristic cell wall substructure consisting of parallel cellulose fibrils arranged in layers in perpendicular orientations [10-12]. Furthermore, phylogenic studies have shown that the family *Oocystaceae* is an independent lineage within Trebouxiophyceae and is closely related to the *Chlorellaceae* [10, 12]. Currently, there are up to 268 species in the family *Oocystaceae* listed in AlgaeBase (http://www.algaebase.org) [10, 12]. Whether these species are taxonomically uniform remains unclear [12]. Additionally, *Oocystis lacustris* and *Oocystis parva* are widely adapted species. While *Oocystis* species are mostly freshwater green algae of the family *Oocystaceae* [13], they are considered a challenging genus with respect to the presence or absence of pyrenoids, and the biological properties of *Oocystis* spp. are very location dependent [14]. For instance, *Oocystis borgei*, a green microalga and common dominant species in subtropical prawn ponds, exhibits a stable population size and strong adaptability to environmental stress conditions [11, 15]. It performs urea-based nitrogen removal in aquaculture wastewater [11, 15]. The freshwater green alga *Oocystis solitaria* has been studied for its potential to remove lithium ions from aqueous solutions [16]. However, there is little information on the establishment of culture conditions for *Oocystis* species and the production of lipids and high value-added metabolites [16]. In addition, a model that describes the relationship between bacteria and *Oocystis* spp. has not yet been developed.

Microalgae are recognized as promising producers of many bioactive products [17]. Of these bioactive compounds, fucose is a useful rare sugar with high economic potential in the pharmaceutical and cosmetic industries because of its anti-cancer, anti-oxidative, anti-inflammatory, anti-hypertensive, and anti-aging activities [18-20]. It is produced through a chemical synthesis process in which other monosaccharides serve as raw materials for its generation [18-20]. In addition, microbial or enzymatic biosynthesis could be another approach for fucose production [21, 22]. The other processes without a biosynthesis step are mostly based on the concept of recovering fucose from the hydrolysate of polysaccharide-containing biomass [5, 23]. As noted, most studies on fucose production have been based on seaweed [23, 24]. However, producing fucose from algae is not yet cost effective enough to compete with other biosynthetic strategies [23].

Eicosapentaenoic acid (EPA; 20:5n-3) is known to be an ω-3 polyunsaturated fatty acid and is considered a potential source of this important compound [25]. EPA performs many vital functions in biological membranes and serves as a precursor of a variety of lipid regulators in cellular metabolism [26]. As a result, EPA can play a critical role in regulating biological functions and in the prevention and treatment of human diseases such as heart disease, hypertension, and inflammation [27, 28]. EPA has been found in a wide variety of microalgae, including diatoms, which contain large quantities of high-quality EPA [26]. In contrast to the large number of known EPA-containing microalgae, only a few microalgal species have exhibited applicable production potential [17, 25]. This has resulted in low algal biomass (or low cell density) following the low specific growth rate of microalgae grown under traditional cultivation conditions [1, 18].

Numerous strategies have been reported for economically mass producing high-value products such as EPA or fucose based on microalgae [1]. These include isolation of high EPA- or fucose-yielding microalgal strains, development of strains by genetic engineering methods such as genome editing and foreign introduction, improvement of optimized culture conditions, and establishment of cost-effective cultivation systems [1, 28, 29]. Economically achieving any of these approaches should be preceded however by screening for an excellent single strain [6, 30]. Producing EPA and fucose by a single species would substantially reduce costs and lead to industrial microalgal production of these two high-value bioactive molecules [31].

The development of an economically practicable access to microalgae production is very important, but requires a thorough understanding of microalgal physiology [32]. Previously, it has been shown that the production of desirable metabolites increases following changes to microalgal culture conditions, particularly nutrient supply [33]. However, maximum biomass productivity and concentration of target products are usually inversely proportional under identical cultivation conditions [34, 35]. Recent studies have demonstrated an increasing interest in the interactions between microalgae and other microorganisms [36]. Greater insight into the modulation of such interactions (naturally present in their growth medium or added), especially involving bacteria, should be useful for promoting the algal biomass production of high-value bioactive metabolites [37]. Little attention has been paid to the controlled use of microalgae-bacteria consortia [37]. However, reports on microalgal-bacterial interplay have highlighted the key roles of mutualism, commensalism, and parasitism in algal growth [38]. Some of these interactions can be found among different microorganism species, while others are strictly species specific [39]. For instance, algal growth has been shown to be enhanced by bacterial growth-promoting factors such as indole-3-acetic acid [40]. Vitamin B_12_ production in algal cultures and bacterial siderophores is also involved in the increase of activated microalgal growth [40-41]. Increases in the intracellular levels of carbohydrates, lipids, proteins, and pigments of microalgae coupled with algal growth enhancers have also been reported [41]. For instance, when the green alga *Chlorella vulgaris* was co-cultured with four growth-promoting bacteria (*Flavobacterium*, *Hypomonas*, *Rhizobium*, and *Sphingomonas*) in a factitious microalgae-bacterial consortium, the final concentration of algal cell biomass and lipid content increased [42]. In this sense, economical algal production might occur in the presence of bacteria, and microalgae-bacterial interactions could aid in producing high-value algal by-products [43].

The consortia formed by *Oocystis* sp. and *Sphingomonas* sp. has also been mostly unnoticed [43]. Hence, in this study we aimed to investigate the impact of this microalgae (*Oocystis* sp.)-microoganism (*Sphingomonas* sp.) interaction on the production of high-value compounds. In brief, we investigated the possibility of EPA and fucose production following the establishment of culture conditions in a new freshwater microalgal strain, *Oocystis* sp. KNUA044, but did not maximize the productivity of these molecules through optimized culture conditions. Based on this, we tested the effects of *Sphingomonas* sp. KNU100-dependent culture strategies for the production and accumulation of EPA and fucose by *Oocystis* sp. KNUA044. We analyzed the content and fatty acid compositions of these two high-value by-products, particularly linolenic acid (C18:3 ω3), as well as the biodiesel quality from the microalgal biomass, and evaluated whether multiple high-value products could be obtained from a single microalgal species by introducing a microalgae-bacteria interaction mode for algal biotechnological applications.

## Material and Methods

### Isolation and Identification of Microalga *Oocystis* sp. KNUA044 and Bacterium *Sphingomonas* sp. KNU100

Freshwater samples were collected at the Chilgok Agricultural Technology Center (36° 02′ 18.91′′N, 128° 22′57.71′′E) in Korea. To isolate the microalgae, 1 ml of freshwater was inoculated into 100 ml of BG–11 medium containing chloramphenicol (30 μg/ml) and ampicillin (100 μg/ml) to prevent bacterial richness and prepare for the growth of an axenic algal cell culture. The inoculated sample was incubated at 25°C with shaking at 160 rpm in a light:dark cycle (16:8 h). Then, the resulting microalgal cells were centrifuged and streaked onto BG-11 agar medium supplemented with the same antibiotics. A single microalgal colony was transferred to R2A agar medium until a pure culture was obtained. For morphological analysis, cultured microalgae cells were visualized at 400× magnification using a light microscope (ZEISS Axio Imager A2; Carl Zeiss, Germany). For molecular identification, genomic DNA was isolated from freshly cultured microalgal cells and amplified using PCR, with each primer set of 18S ribosomal DNA and internal transcribed spacers (ITS), as reported previously [45, 46]. Each PCR amplicon was ligated to pGEM Easy Vector (Promega, USA) and transformed into *Escherichia coli* DH5α. The resulting plasmid DNA was sequenced and identified using the NCBI BLAST tool. A phylogenetic tree was constructed using maximum likelihood (ML) with 1,000 bootstrap replicates [44].

For identification of prokaryotic organisms, bacteria combined with the isolated microalgal strain were picked and cultured for 24 h at 25°C with shaking (180 rpm) in BD Difco R2A broth medium (Thermo Fisher Scientific, USA) containing G418 (geneticin; 400 μg/ml). The cultured solution was diluted with R2A broth medium and spread onto the same R2A medium plus 1.5% agar. Genomic DNA was isolated from bacterial cells grown in R2A broth medium containing a single colony. The PCR amplicon was ligated and sequenced using a commercial primer set of 16S rDNA. Species identification was performed using the NCBI BLAST tool [45, 46].

### Culture Conditions for the *Sphingomonas* sp. KNU100–Dependent *Oocystis* sp. KNUA044 Strain

First, to identify the culture medium, algal strains were cultured on various media including BG–11 [47], R2A broth [48], BBM [49], and OHM [50] in 250 ml flasks. Based on these results, subsequent experiments were performed as follows: The isolated algal strain was cultured in a commercial BG–11 solution (Sigma Aldrich, USA) containing a clear supernatant of *Sphingomonas* sp. KNU100 strain culture solution for a specified time (*i.e.*, 12, 24, 36, and 48 h) at 25°C in R2A medium. To completely remove the bacteria, the cultured solution was centrifuged at 8,000 ×*g* for 20 min at 25°C, and then the clear supernatant was filtered through a Corning syringe filter (0.22 μm pore size; Corning Inc., USA). For photoautotrophic cultivation, the algal strain was inoculated into a mixed solution containing 2 ml of BG–11 solution (adjusted to 1×) and 98 ml of a clear supernatant of bacterium solution, and cultured at 25°C with shaking (150 rpm) under a light:dark cycle (16:8 h) and light intensity (approximately 70–100 μmol/m^2^/s). Optical density was measured at 680 nm using a spectrophotometer (2120 UV; Mecasys, Korea). Algal cells were counted every 2 days using a light microscope (ZEISS Axio Imager A2; Carl Zeiss) at 400× magnification with a Neubauer-improved cell counting chamber according to the manufacturer´s instructions (Paul Marienfeld GmbH & Co. KG, Germany).

### Determination of Total Carbohydrates in Algal Biomass

Carbohydrates were determined by using phenol-sulfuric acid as reported previously [51]. Freeze-dried algal biomass (10 mg) was reconstituted in water (10 ml) to prepare a known sample concentration for each sample (1 mg/ml). Aliquots (1 ml) were reacted with 3 ml of sulfuric acid (72 wt%) and 1 ml phenol (5%, w/v) in a boiling water bath. The mixtures were incubated for 5 min at 90°C and cooled at room temperature. The absorbance was measured at 490 nm using a spectrophotometer. Total carbohydrate content was calculated from a standard curve based on glucose. Next, monosaccharide quantification from polysaccharide in carbohydrates was determined according to a reduced-scale hydrolysis procedure, based on the NREL Laboratory Analytical Procedure [52]. In brief, approximately 50 mg of lyophilized algal biomass was subjected to two-stage sulfuric acid hydrolysis (1 h at 30°C in 72 wt% sulfuric acid, followed by 1 h at 121°C in 4 wt% sulfuric acid in an autoclave). After hydrolysis, the acid-insoluble residue was separated from the hydrolysate using ceramic filtering crucibles. Soluble carbohydrates (glucose, galactose, fucose, mannitol, and sorbitol) were determined by high-performance liquid chromatography with refractive index detection (HPLC-RID).

### Determination of Total Lipid Content and Fatty Acid Composition in Algal Biomass

Freeze-dried samples of microalgae biomass (10 mg) were suspended in 1 ml distilled water. Aliquots (100 μl) of the suspended solution were reacted with 2 ml of sulfuric acid (98%), heated for 10 min at 100°C in a water bath, and cooled for 5 min in an ice bath. Next, 5 ml of freshly prepared phosphor-vanillin reagent was added, and the sample was incubated for 15 min at 37°C with shaking (200 rpm). Absorbance was measured at 530 nm to quantify the lipid within the sample. Lipid content was calculated using a commercial canola oil-based (final concentration of 2 mg/ml) standard curve. The standard lipid stock was prepared using a commercial canola oil at 20 mg in 10 ml chloroform [53].

For fatty acid composition, approximately 30 mg of lyophilized algal biomass sample was prepared as previously reported [54]. The resulting fatty acid methyl ester was analyzed by gas chromatography mass spectrometry (GC-MS) (Agilent 7890A; DB-FFAP 30 m 0.25 mm i.d. and 0.25 μm film thickness). The initial temperature of 50°C was maintained for 1 min. The temperature was increased to 200°C at a rate of 10°C/min for 30 min, then increased to 240°C at a rate of 10°C/min, and held for 20 min. The injection volume was 1 μl with a split ratio of 20:1. Helium gas was supplied at a constant flow rate of 1 ml/min. The quantitative fatty acid composition was determined by comparing the retention time of the peaks with the Wiley/NBS libraries as an internal standard. The quantitative composition was obtained by area normalization and expressed as a mass percent [29].

### Ultimate Analysis of Algal Biomass 

Ultimate analysis was carried out using an elemental analyzer (Perkin Elmer 2400; PerkinElmer Inc., USA) to determine the concentration of carbon (C), hydrogen (H), nitrogen (N), and sulfur (S). Protein content was determined by multiplying the N content by a factor of 6.25 [55]. The calorific value (CV) was calculated as follows: CV = 0.3278C + 1.4149H + 0.09257S – 0.1379O + 0.637, where C, H, O, and S represent carbon, hydrogen, oxygen, and sulfur in mass percentages (wt%), respectively [56].

### Estimation of Biodiesel Quality of Algal Biomass

The quality of biodiesel was determined by assessing the saponification value (SV), iodine value (IV), degree of unsaturation (DU), cetane number (CN), cold filter plugging point (CFPP), oxidation stability (OS), kinematic viscosity (υ), and density (ρ), which were calculated based on the fatty acid composition using empirical equations, as reported previously [57].

### Statistical Analysis

Significant differences between mean values were assessed by one-way analysis of variance (ANOVA). For all data analyses, a level of *p* < 0.05 was considered statistically significant. Where significant differences were observed, treatment means were differentiated using pairwise comparison by applying Tukey’s test.

## Results and Discussion

### Identification of Microalga *Oocystis* sp. KNUA100 and Bacterium *Sphingomonas* sp. KNU100

**Fig. 1:**
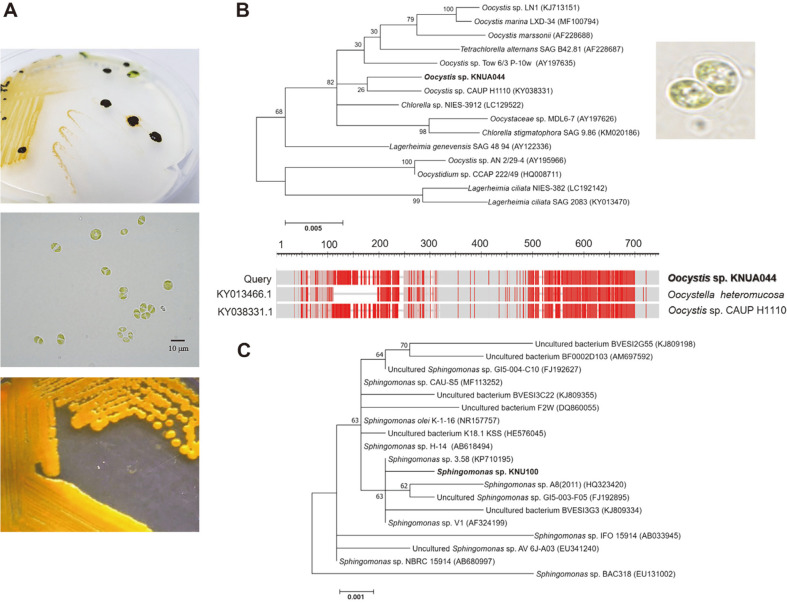
Isolation and identification of *Oocystis* sp. KNUA044 and *Sphingomonas* sp. KNU100. (**A**) The coexisting morphology of purely isolated axenic microalga *Oocystis* sp. KNUA044 and a single bacterium *Sphingomonas* sp. KNU100 grown in R2A agar medium is displayed in the upper panel. The morphology of microalga *Oocystis* sp. KNUA044 strain grown in BG–11 medium in the presence of *Sphingomonas* sp. KNU100 strain culture solution is shown in the middle panel. The scale bar indicates 10 μm. The colony morphology of *Sphingomonas* sp. KNU100 strain grown in R2A agar medium is presented in the lower panel. (**B**) Identification of the algal strain *Oocystis* sp. KNUA044 using primer sets corresponding to the 18S rDNA (upper panel) and ITS region (lower panel) at the molecular level. Phylogenetic analysis of 18S rDNA and ITS gene sequences. Topology represents the best ML tree. A new sequence of *Oocystis* sp. KNUA044 strain is highlighted in bold. Numbers at the branches indicate bootstrap support based on maximum likelihood (ML) and Bayesian posterior probabilities (BI). Support ≥50% for ML and ≥0.95 for MB is shown in ML/BI. (**C**) Molecular identification of the *Sphingomonas* sp. KNU100 strain using a well-known 16S rDNA primer set. Phylogenetic analysis of the 16S rDNA gene sequence was performed as described for the *Oocystis* sp. KNUA044 strain.

Pure isolated algal and bacterium strains were detected on R2A agar medium (Fig. 1A, upper panel). Many algal culture collections maintain a symbiotic relationship between the algal isolates and associated bacteria, suggesting that an algal isolate often contains one or more bacteria species [39]. To purely isolate algal strains, each algal and bacterial colony was cultured in BG–11 and R2A media, respectively, and then strain identification was performed. A taxonomic approach to identify algal species was conducted by combining morphological, molecular, and phylogenetic methods. Cells in solitary or 2–4-celled colonies were elliptical to cylindrical, with round ends and sometimes tapered thickness, approximately 6.5–12.0 μm long and approximately 3.0–6.0 μm wide (Fig. 1A, middle panel). According to a previous report, *Oocystis* sp. can exist as detached cells, arranged in 2–, 4–, 8–, 16– and 32–celled groups or coenobia, or tied by pseudofilaments [58]. The cells are egg- or spindle-shaped, sometimes globular, or asymmetrical [58]. One, a few, or numerous chloroplasts are present in a single cell, parietal or nearly so, with a pyrenoid that is sometimes not clearly detected [58]. The cell wall is tender, with or without thickened terminals, or covered with spines or granules [58].

For molecular analysis, 18S rDNA and ITS sequences were obtained for the algal strain. Partial sequencing of the 18S rDNA PCR product produced a 1,767 bp sequence, while the PCR amplicon of the ITS sequence produced approximately 426 bp. The sequence similarity of the algal strain 18S rDNA and ITS was 98.72% and 88.39% that of *Oocystis* sp., respectively (Table 1). The final alignment of the 18S rDNA and ITS was positioned in one of the *Oocystis* clusters and formed a well-defined freshwater clade with the other *Oocystis* spp. strains (Fig. 1B). The genus *Oocystis* proved to be paraphyletic and some species were precluded from *Oocystaceae*, while a few other species were newly redefined as members of this family [58]. The most controversial discovery of the molecular phylogeny was the polyphyletic status of the morphologically well-defined *Oocystis* [58]. Only four sequences of *Oocystis* species have been resolved so far [58]. Taxonomical classifications in the genera *Neglectella*, *Oocystidium*, *Oocystis*, and Ooplanctella were formed based on coincident molecular and morphological results [58]. Therefore, the algal strain was named *Oocystis* sp. KNUA044.

The isolated bacterium produced yellow-pigmented colonies when grown on R2A agar medium (Fig. 1, lower panel). The PCR product of 16S rDNA generated 1,449 bp, which was 99.72% similar to *Sphingomonas* spp.(Table 1), as was clearly shown by sequence analysis. In particular, *Sphingomonas* sp. KNU100 had high sequence homology to *Sphingomonas* spp., as shown in Fig. 1C. *Sphingomonas* comprises gram-negative, off-white, yellow-or orange-pigmented, and rod-shaped bacteria [59].

### Establishment of *Sphingomonas* sp. KNU100-Dependent *Oocystis* sp. KNUA044 Culture Conditions

*O. solitaria* was grown in axenic cultures at 24°C ± 2°C under a continuous illumination intensity of 48.6 mol/m^2^/s in BG–11 medium for a 12–day incubation period [16]. Unlike *O. solitaria*, *Oocystis* sp. KNUA044 strain was isolated in the summer season under a microalgae mass cultivation system at Chilgok Agricultural Technology Center in Korea. Summer weather is very similar to the light/dark cycle (16-h light/8-h dark cycle) of microalgal culture. The mean water temperature in the summer season was 25.4°C (data not shown). The separation of microalgae, including the *Oocystis* sp. KNUA044 strain, is very important when it matches the separated environmental conditions. On the other hand, microorganisms such as *Sphingomonas* spp. are scarcely affected by the light/dark cycle. For this reason, the isolated indigenous microalga KNUA044 strain was cultured under a light/dark cycle rather than under continuous illumination.

In microalgae-bacteria consortia, many bacteria are of the same genera as those found in natural algal environments [39]. Based on this fact, we tried to establish a platform for *Oocystis* sp. KNUA044 culture conditions. As shown in Table 2, the algal strain showed poor growth under various culture media containing BG–11 supplemented with different nitrogen sources (NaNO_3_, NH_4_Cl, and urea adjusted to 250 mg/l of final concentration), R2A, OHM, and BBM. Next, we assumed that *Sphingomonas* sp. KNU100 strain could enhance the growth of the algal *Oocystis* sp. KNUA044 strain, since the algal strain was identified in the presence of the bacterium on R2A agar medium (Fig. 1, upper panel). As expected, addition of *Sphingomonas* sp. KNU100 culture solution to R2A medium with 24 h incubation improved growth development of the algal strain in BG-11 medium (Table 2). At this point, microalgal growth in different media should be compared scientifically and expressed numerically. However, as information on establishing culture methods of *Oocystis* strains including KNUA044 is limited, it is very difficult to do so. Hence, the results were expressed as good or bad depending on whether growth was shown or not.

**Fig. 2:**
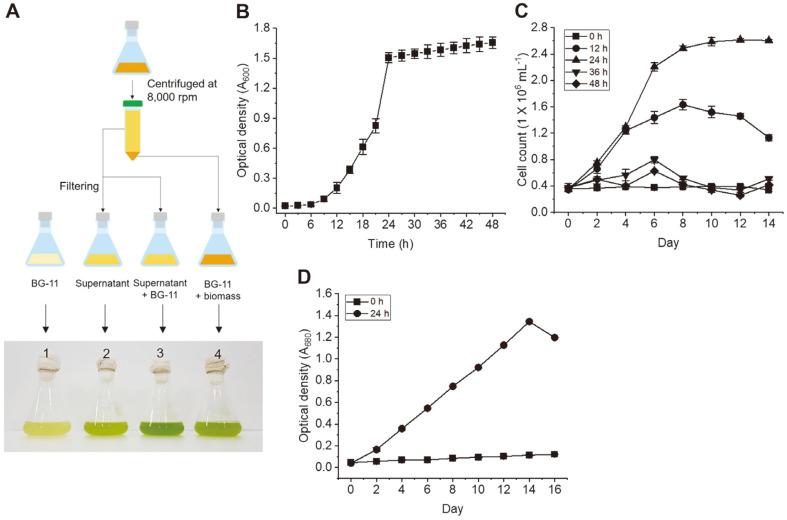
Enhancement of algal biomass in *Oocystis* sp. KNUA044 strain in the presence of a clear supernatant derived by cultivation of the *Sphingomonas* sp. KNU100 strain. (**A**) Establishment of a cultivation platform for the *Oocystis* sp. KNUA044 strain. A clear supernatant solution of *Sphingomonas* sp. KNU100 strain grown in R2A medium was harvested by centrifugation, filtered through a membrane filter (0.2 μm pore size), and then aliquoted into BG–11 medium. The mixture contained 2 ml of a clear supernatant solution of *Sphingomonas* sp. KNU100 strain and 2 ml of BG–11 stock solution (50×), added to 100 ml with distilled water. 1. Solution of BG–11 medium alone; 2. Solution of clear supernatant of *Sphingomonas* sp. KNU100 strain; 3. Mixture solution containing a clear supernatant and BG–11 medium; 4. Solution of bacterial biomass resuspended in BG-11 medium. (**B**) The growth kinetics of the *Sphingomonas* sp. KNU100 strain were analyzed by measuring the optical density at 600 nm using a spectrophotometer. Algal growth kinetics was determined by counting the cell number (**C**) and measuring the absorbance at 680 nm (**D**). At this time, *Oocystis* sp. KNUA044 strain was cultured in freshly prepared BG–11 medium supplemented with a clear solution of *Sphingomonas* sp. KNU100 strain grown in R2A medium for 0 h (square), 12 h (circle), 24 h (up triangle), 36 h (down triangle), and 48 h (diamond).

Next, we analyzed algal growth kinetics in three classes of bacterial culture including clear supernatant, bacterial biomass, and a combined solution of supernatant and bacterial biomass, under the same conditions. First, *Sphingomonas* sp. KNU100 was incubated in R2A broth for the indicated time at 25°C until optical density at 600 nm reached approximately 1.5. The cleared bacterium supernatant was mixed in BG-11 medium after centrifugation, as described in Materials and Methods. Growth of *Oocystis* sp. KNUA044 was determined by direct counting with a hemocytometer under a microscope. In addition, the microalgal cell density was measured by the optical density of a clear algal solution at 680 nm after centrifugation. *Oocystis* sp. KNUA044 grew better on the BG–11 medium combined with the cleared supernatant without bacterial biomass (6.84 × 10^5^ cells/ml) as compared to BG–11 (2.15 × 10^5^ cells/ml), cleared bacterium supernatant solution (3.62 × 10^5^ cells/ml), and *Sphingomonas* sp. biomass solution suspended in BG–11 medium (4.54 × 10^5^ cells/ml). The algal strain displayed a higher growth rate in BG–11 medium containing clear supernatant solution compared to other culture conditions based on BG–11. BG–11 medium without any bacterial culture solution showed the poorest growth (Fig. 2A). The medium composition was a mixture of 2 ml BG–11 medium (50× stock solution) and 98 ml of *Sphingomonas* sp. KNU100 strain culture solution grown in R2A medium. Next, we analyzed the growth of the algal strain according to the culture time of the *Sphingomonas* sp. The growth kinetics of the *Sphingomonas* sp. KNU100 strain is shown in Fig. 2B. The bacterial strain showed approximately 12–24 h of exponential phase, and then entered the stationary phase. For the *Oocystis* sp. KNUA044 strain, a clear solution of *Sphingomonas* sp. KNU100 strain cultured for 24 h at 25°C exhibited excellent cell number-dependent growth of the algal strain compared to that of the culture solution for 12, 36, and 48 h (Fig. 2C). Additionally, the exponential phase of the algal strain was identified at day 14 post cultivation, and the specific growth rate (*μ*_max_) was 0.16 ± 0.01/day. The growth of microalgae culture differs between algal species and is dependent on the photoperiod. For instance, the *μ*_max_ value of fucose production-capable *Botrycoccus braunii* was 0.05–0.17 [60, 61]. Compared to other microalgae, the growth of *Oocystis* sp. KNUA044 strain was slow. In this study, optimized culture conditions for the KNUA044 strain were not fully established. However, one purpose of this study was to establish the culture conditions for the *Oocystis* sp. KNUA044 strain, as little information on the subject is available. In the future, we intend to maximize the production of algal biomass by establishing the optimal culture conditions.

Despite this dearth of information on *Oocystis* spp. culture conditions, an exception is *O. solitaria*, as discussed above. The unicellular green alga *O. submarina* was identified microscopically as the sole organism causing algal bloom, and a high density of *Oocystis*-associated purple bacteria including *Loktanella vestfoldensis*, *Roseinatronobacter* sp., and *Rhodobaculum claviforme* was observed in the bottom layer [62]. On the other hand, members of *Sphingomonas* are of biotechnological interest owing to their ability to degrade environmental pollutants such as xenobiotics as well as their potential to produce useful high-value products such as exopolysaccharides and carotenoids [59]. Hence, our results suggest that the culture conditions for the *Oocystis* sp. KNUA044 strain were BG–11 medium supplemented with a clear solution of the *Sphingomonas* sp. KNU100 strain grown for 24 h.

Microalgae-bacteria consortia are often considered detrimental to algal growth [33]. However, recent studies have shown that algal interactions not only promote algal growth, but also provide advantages in downstream processing for algal biotechnological applications [33, 63]. The interaction of microalgae and other microorganisms greatly increases the efficiency of algal biomass production and its chemical composition [33]. As shown in our results obtained from the *Sphingomonas* sp. KNU100 strain, heterotrophic bacteria synthesize important compounds for growth stimulation, morphogenesis, and abiotic and biotic resistance against environmental stresses such as high salinity and temperature [63, 64]. These compounds include signaling and transporter molecules, micro- and macronutrients such as carbon, sulfur, nitrogen, and phosphorous, siderophores (*e.g.*, iron-siderophores to bind iron), growth stimulants such as indole acetic acid and vitamins B [vitamins B_12_ (cobalamin), B_1_ (thiamine), and B_7_ (biotin)], primary metabolites such as amino acids, phytohormones, volatile compounds (VCs), antibiotics, and quorum sensing molecules [63-66]. Many studies have reported microalgal growth promotion by providing vitamins and growth-promoting compounds from bacteria [33, 63, 64]. However, *Sphingomonas*-mediated heterotrophic microalgae interaction is still unknown. Recently, volatile indoles of *Sphingomonas* sp.-derived VCs have been shown to play a critical role as functional agents that enhance growth (10–70%; up to 3.31 g/l) and production of lipids (22–28%) and triacylglycerol (20%) in *C. vulgaris* [67]. However, there are pertinent questions related to the mechanism of such interactions.

On the other hand, macronutrients including nitrogen, phosphorous, and sulfur are fundamental factors required to produce organic matter from inorganic carbon through photosynthesis in microalgae [33]. Lower concentrations of these components can cause decreased algal cell growth [33]. Some of these elements are discovered in nature in a chemical form that cannot be synthesized by algal cells [33]. Bacteria are capable of fixing atmospheric nitrogen, solubilizing phosphorus, and producing plant hormones (auxins, gibberellins, and cytokinins) and signaling molecules (ethylene, nitrite, and nitric oxide) [63, 66]. As a result, these macronutrients can be metabolized by microalgae [33, 63, 66]. Therefore, we hypothesize that a complex physiological relationship might exist between *Oocystis* sp. KNUA044 and *Sphingomonas* sp. KNU100, and that this relationship includes a wide range of released metabolites whose functions need to be further verified. A potential employment of bacterium metabolites in microalgal cultivation will have a positive effect on *Oocystis*-based biotechnological applications.

### Biochemical Properties of Algal Biomass in *Oocystis* sp. KNUA044

Algal biomass cultured for 14 days was harvested, freeze-dried, and used for subsequent experiments. The characteristics of the algal biomass are presented in Table 3. Biomass productivity and total carbohydrate productivity were 28.57 ± 2.97 and 8.33 ± 0.84 mg/l/day, respectively. The carbohydrate, protein, and lipid content was 29.4 ± 2.2, 43.9 ± 0.3, and 13.9 ± 0.6 wt%, respectively. The diatom microalga *Phaeodactylum tricornutum* has the ability to produce fucose containing approximately 36.4 wt% crude protein, 26.1 wt% carbohydrate, and 18.0 wt% lipid [68]. Microalgae are capable of producing biofuel by accumulating lipids and carbohydrates as major energy storage molecules. In contrast, owing to their high protein content, microalgae are considered suitable as feedstocks for food and feed production for human and animal nutrition, rather than for biofuel production [63].

In the ultimate analysis, carbon (C), hydrogen (H), nitrogen (N), oxygen (O), and sulfur (S) content was 47.6 ± 0.7, 6.8 ± 0.2, 7.0 ± 0.1, 37.9 ± 0.9, and 0.7 ± 0.1 wt%, respectively. The CV was 19.8 ± 0.4 MJ/kg. Based on the high C content, polysaccharide algal biomass was analyzed. The concentrations of glucose, galactose, fucose, mannitol, and sorbitol were 7.1, 16.7, 3.3, 0.7, and 0.7 mg/100 mg, respectively. In particular, the algal strain *Oocystis* sp. KNUA044 produced 3.3 mg/100 mg of fucose. This represented fucose productivity of 0.94 ± 0.09 mg/l/day. The low fucose productivity of *Oocystis* sp. KNUA044 was because the optimal culture conditions have not yet been established. Nevertheless, our results are meaningful in that we discovered the ability of *Oocystis* sp. KNUA044 strain to produce fucose. To date, there has been limited knowledge about the relationship between *Oocystis* sp. and fucose. Previously, fucose was found only in the diatoms *P. tricornutum* [68], *Nostoc microscopicum* [68], *B. braunii* (6–463 mg/l) [61], and *Graesiella* sp. [69] in very low concentrations. Among macroalgae, *Himanthalia elongata*, has the highest fucose content (28.7 ± 2.6 g/kg), followed by *Laminaria ochroleuca* (24.9 ± 0.3 g/kg) and *Undaria pinnatifida* (7.3 ± 0.4 g/kg) [7]. Fucoidans or fucans are naturally occurring L–fucose sulfated polysaccharides that are typically found in the cell walls of brown algae, but not green algae. Fucoidans have several health-promoting properties such as anti-oxidant, anti-bacterial, anti-obesity, anti-tumor, anti-inflammatory, anti-viral, and immunomodulatory activities [7, 68]. As a result, the green microalgae *Oocystis* sp. KNUA044 is a potential source of these health-promoting molecules, even though fucose production is dependent on the origin of the alga.

### Fatty Acid Composition and Biodiesel Quality of Algal Biomass

Fatty acid profiling and biodiesel parameters were also examined in the freeze-dried algal biomass. As shown in Table 4, the content of saturated fatty acids (SFAs), monounsaturated fatty acids (MUFAs), and polyunsaturated fatty acids (PUFAs) was 29.41, 9.90, and 60.68 wt%, respectively. In relation to SFAs, C14:0, C15:0, C16:0, C17:0, and C18:0 content was 0.98, 1.06, 24.87, 1.19, and 1.32 wt% of total fatty acids (FAs), respectively. For MUFAs, C16:1 ω7 and C18:1 ω9 levels were 6.95 and 2.95 wt%, respectively. For PUFAs, C16:2, C16:3 ω3, C18:2 ω6, C18:3 ω3, and C20:5 ω3 content was 2.21, 10.08, 2.05, 40.27, and 6.07 wt%, respectively. The dominant fatty acid among SFAs was C16:0 (palmitic acid; 24.87 wt%), whereas the dominant fatty acids among PUFAs were C18:2 (linoleic acid; 10.08 wt%) and C18:3 (linolenic acid; 40.27 wt%). As noted above, co-cultivation of axenic *C. vulgaris* with four different growth-enhancing bacteria (*Flavobacterium*, *Hypomonas*, *Rhizobium*, and *Sphingomonas*) revealed a symbiotic relationship in an artificial microalgae-bacteria consortium [42]. Fatty acid profiling analysis of the biomass obtained from the algae-bacteria co-culture showed a significant increase in oleic (C18:1) and palmitic (C16:0) acids, based on fatty acid composition during the axenic cultivation of *C. vulgaris*, which is dominated by hexadecatrienoic (C16:2) and linoleic (C18:2) acids [67].

As shown in Table 4, these essential high-value long-chain PUFAs (LC–PUFAs), including C18:3 ω3, C18:2 ω6, and C20:5 ω3, which are beneficial to human health, can be produced by some microalgal species such as *Crypthecodinium cohnii*, *Nannochloropsis oceanica*, and *P. tricornutum* [5]. However, to date, there have been no reports of *Oocystis* sp. in relation to LC–PUFAs. The level of PUFAs was approximately 1.8-fold higher than that of SFAs and MUFAs (Table 4). Importantly, PUFAs have demonstrated protective and curative activities against inflammatory, diabetes and Alzheimer’s disease [70]. In addition, PUFAs are positively related to myelin integrity in patients with depression [71]. In microalgae, the average lipid content varies from 1 to 70% (w/w) and depends on the species, life cycle, and cultivation conditions including the nutritional and environmental requirements of the microalga [1, 70].

*Oocystis* sp. KNUA044 produced 6.07 wt% of C20:5 ω3 (EPA) of total FAs. Omega–3 (n–3) LC–PUFA EPA is a marine-based omega–FA and is an essential FA component with various human health benefit applications [1]. In addition, EPA can be effective in preventing or treating many diseases [5, 72]. For instance, an intake rich in EPA can reinforce cancer treatments by enhancing the immune response to therapies [71]. Currently, EPA is mainly produced from marine fish oil and fishmeal [73], but algal *Nannochloropsis* spp. are interesting as an alternative source because they can produce EPA to 1.1–12% of their dry weight [73]. *P. tricornutum* can also accumulate EPA [68]. Thus, our results indicate that the blue-green alga *Oocystis* sp. KNUA044 could be used for the production of high value-added by-products such as linolenic acid and EPA. Therefore, this study is of great significance for understanding the physiological characteristics of *Oocystis* sp. KNUA044 strain for the production of high-value by-products such as LC–PUFAs including EPA. Although microalgae containing fucose and EPA are rare, *Oocystis* sp. KNUA044 can produce fucose and EPA. As noted above, fucose or fucoidan is a high-value monosaccharide or polysaccharide of carbohydrate and has potential medical applications including anti-cancer properties [18, 30]. EPA also plays a key role in the prevention and treatment of various human diseases such as cancer, obesity, rheumatoid arthritis, diabetes, and Alzheimer’s disease [18, 30, 71]. Altogether, the highly valuable compounds derived from *Oocystis* sp. KNUA044 mean this microalgal strain is a potential candidate for pharmaceutical and cosmetic applications.

For biodiesel quality, the physicochemical properties of biodiesel obtained from *Oocystis* sp. KNUA044 strain exhibited low kinematic viscosity (υ; 3.4 g/cm^3^), low density (ρ; 0.89 mm^2^/s), low CN (36.5), high IV (176.3 g I2/ 100 g fat), high OS (5.4 h), low CFPP (–6.6°C), and high C18:3 content (40.2 wt%). The SV and DU values were 199.3 mg KOH/g and 131.3, respectively (Table 5). Among the parameters analyzed, high IV, low CN, and high C18:3 content did not meet major biodiesel specifications of American (ASTM D6751) and European Standard Organization (EN 14214) standards. The low CN value could be attributed to the average amounts of SFAs and MUFAs (39.31 wt%), as shown in Table 4, which lead to poor combustion, generating engine motor inefficiency and increasing nitrogen oxides in exhaust emissions [74]. Higher IV values following high content of PUFA and MUFAs (70.58 wt%) may result in the formation and accumulation of glycerides and deposition of lubricant in the engine [74]. Thus, our results show that the microalga *Oocystis* sp. KNUA044 strain has potential as an alternative feedstock to produce high-value bioproducts other than biofuel.

## Conclusion

The new freshwater microalgal *Oocystis* sp. KNUA044 was cultured in BG–11 medium in the presence of the cleared supernatant of the bacterium *Sphingomonas* sp. KNU100. Cultured algal biomass was effective for production of high-value bioactive compounds rather than biofuel production. Thus, our results suggest that a consortium of bacterium *Sphingomonas* sp. KNU100 and microalgae *Oocystis* sp. KNUA044 could be utilized to increase algal biomass following enhanced algal cell growth and to produce high-value compounds including fucose, linolenic acid (C18:3 ω3), and EPA (C20:5 ω3). These high-value bioactive compounds and metabolites can be utilized in a wide range of industrial applications in the medical, pharmaceutical, and cosmetics industries. Currently, our knowledge of the microalgae-bacterium interaction is very limited. In the future, understanding the interaction between the *Oocystis* sp. KNUA044 strain and *Sphingomonas* sp. KNU100 strain could lead to identification of unresolved medium ingredients that could be provided by bacterium cultivation. Furthermore, the biological enhancers provided by bacterial metabolites could also reduce the necessity for external requirements in relation to microalgal growth.

## Figures and Tables

**Table 1:** Identification of bacteria and microalgae using molecular markers.

Marker gene	Product size (bp)	Closet match (GenBank number)	Sequence similarity (%)
16S rDNA	1,449	*Sphingomonas* sp. 3.58 (KP710195)	99.72
18S rDNA	1,767	*Oocystis* sp. CAUP H1110 (KY038331)	98.72
ITS	426	*Oocystis heteromucosa* CB_210 (KY013466)	88.39

**Table 2:** Culture tests in various media to facilitate the growth of the *Oocystis* sp. KNUA044 strain.

Medium	Culture type	Growth
BG–11	Photoautotroph	Bad
BG–11 (NaNO_3_)	Photoautotroph	Bad
BG-11 (NH_4_Cl)	Photoautotroph	Bad
BG-11 (urea)	Photoautotroph	Bad
BG-11 (NH_4_Cl + NaHCO_3_)	Photoautotroph	Bad
R2A	Photoautotroph	Bad
OHM	Photoautotroph	Very bad
BBM	Photoautotroph	Very bad
BG–11 + *Sphingomonas* sp. KNU100 culture solution	Photoautotroph	Good

**Table 3:** Characteristics of algal biomass in the *Oocystis* sp. KNUA044 strain.

Cellular component	Carbohydrate (wt%)		Protein (wt%)		Lipid (wt%)	

	29.4 ± 2.2		43.9 ± 0.3		13.9 ± 0.6	

Ultimate analysis	C (wt%)	H (wt%)	N (wt%)	S (wt%)	O (wt%)	CV (MJ/kg)

	47.6 ± 0.7	6.8 ± 0.2	7.0 ± 0.1	0.7 ± 0.1	37.9 ± 0.9	19.8 ± 0.4

Monosaccharide (mg/ 100 mg)	Glucose	Galactose	Fucose	Mannitol	Sorbitol	

	7.1	16.7	3.3	0.7	0.7	

Productivity (mg/L/d)	Biomass		Fucose		Total carbohydrate	

	28.57 ± 2.97		0.94 ± 0.09		8.33 ± 0.84	

**Table 4:** Fatty acid profiling of algal biomass.

Fatty acid	Composition (wt%)
C14:0	0.98
C15:0	1.06
C16:0	24.87
C16:1 ω7	6.95
C16:2	2.21
C16:3 ω3	10.08
C17:0	1.19
C18:0	1.32
C18:1 ω9	2.95
C18:2	2.05
C18:3 ω3	40.27
C20:5 ω3	6.07
SFAs	29.41
MUFAs	9.90
PUFAs	60.68

**Table 5:** Quality of biodiesel derived from algal biomass.

Biodiesel parameters	Value	EN 14214	ASTM D6751
SV (mg KOH/g)	199.3		
IV (g I_2_/100 g fat)	176.3	≤ 120	
CN	36.5	≥ 51	≥ 47
DU	131.3		
CFPP (°C)	–6.6	5≤/≤–20	
Oxidation stability (h)	5.4	≥ 6	≥ 3
Kinematic viscosity (g/cm^3^)	3.4	3.5 – 5.0	1.9 – 6.0
Density (mm^2^/s)	0.89	0.86 – 0.90	0.82 – 0.90
C18:3 (wt%)	40.27	≤ 12	
